# Polypharmacy and patterns of medication use in the final year of life among people living with dementia in Canada: A case-control study

**DOI:** 10.1177/03000605261487270

**Published:** 2026-09-27

**Authors:** Jasmine Mah, Catherine Richer, Abby Emdin, Kenneth Rockwood, Samuel Stewart, Melissa K Andrew, Shanna C Trenaman

**Affiliations:** 1 Division of Geriatric Medicine, Department of Medicine, 3688 Nova Scotia Health, Canada; 2 Department of Family Medicine, 5620 McGill University, Canada; 3 Dalla Lana School of Public Health, 7938 University of Toronto, Canada; 4 Community Health & Epidemiology, 3688 Dalhousie University, Canada; 5 Department of Medicine (Division of Geriatric Medicine), 3688 Dalhousie University, Canada; 6 College of Pharmacy, Faculty of Health, 3688 Dalhousie University, Canada

**Keywords:** Polypharmacy, hyperpolypharmacy, long-term care, dementia, end of life, deprescribing, public health

## Abstract

**Objective:**

At the end of life, changing healthcare needs may lead to decreased use of preventive therapies and increased use of medications for symptom management. This study compared polypharmacy, hyperpolypharmacy, and medication-dispensing patterns among community-dwelling older adults with dementia, community-dwelling older adults without dementia, and older adults with dementia in long-term care.

**Methods:**

We completed a retrospective, matched case-control study using linked administrative health data from Nova Scotia, Canada. We compared polypharmacy, hyperpolypharmacy, and medications dispensed among three groups (community-dwelling older adults with dementia, community-dwelling older adults without dementia, and older adults with dementia in long-term care). We chose community-dwelling older adults with dementia with dates of death between 1 January 2019 and 1 January 2021, and then matched with replacement to community-dwelling older adults without dementia and older adults with dementia in long-term care based on age, administrative sex, and date of death ±30 days. Descriptive comparisons of polypharmacy, hyperpolypharmacy, and medications used during each of three equal periods of the last year of life (early, mid, and late) were made. Logistic regression was used to estimate the probability of polypharmacy and hyperpolypharmacy, adjusting for demographic and clinical factors.

**Results:**

Each group included 1274 individuals. The mean age was 84.8 years. Overall, 50.9% (1944/3822) experienced polypharmacy and 8.7% (332/3822) hyperpolypharmacy, with community-dwelling older adults with dementia consistently having the highest rates. The most common medications dispensed in the last year of life were furosemide, levothyroxine sodium, salbutamol, and vitamin B12.

**Conclusions:**

Community-dwelling older adults with dementia had the highest rates of polypharmacy and hyperpolypharmacy across all time periods assessed. Medications most frequently dispensed in the last year of life did not change in ways that would be expected if deprescribing recommendations were being followed. This suggests that prescribing and deprescribing at the end of life are potential areas for pharmacist support and involvement.

## Introduction

The prevalence of dementia is increasing, with the number of Canadians living with dementia projected to triple between 2020 and 2050 despite declining incidence.^1^ This trend reflects improved survival among people with dementia, attributable to advances in the management of multimorbidity (often pharmacological), stronger social support networks, access to long-term care (LTC) homes, and improved public health measures.^2–4^ Even so, these advances do not reduce the challenges experienced during the last year of life with dementia, which often remains a challenging time for all concerned.^5^ Escalating care needs and increasing multimorbidity contribute to high healthcare utilization during this period. A retrospective Australian study using linked health administrative data demonstrated that, compared with peers without dementia, people with dementia experienced higher rates of urgent healthcare visits, ambulance use, emergency department presentations and associated costs, and short-stay hospital admissions.^6^ Similarly, a US study showed that, in the final year of life, people with neurodegenerative diseases had greater odds of emergency department use than people with malignant brain tumors.^7^

Increased healthcare use during the final year of life is unlikely to be explained solely by dementia. Among older adults, medication use, particularly the use of multiple medications (polypharmacy), is consistently associated with adverse clinical outcomes and greater healthcare.^8^ Usually, we use the term polypharmacy to refer to multiple medications being used concurrently. Polypharmacy is commonly defined as taking five or more medications, whereas hyperpolypharmacy is considered taking 10 or more medications.^9^ Over time, the concept of polypharmacy has evolved to encompass more than medication count; it now includes the clinical appropriateness of prescribing in relation to comorbidities and goals of care.^9^ Among people with dementia, the prevalence of polypharmacy may be as high as 81.5%,^10^ with hyperpolypharmacy affecting approximately 17.6%.^11^ In people with dementia, polypharmacy is associated with greater functional decline,^12^ sedation, emergency department visits (hazard ratio (HR) = 1.02, 95% confidence interval (CI): 1.02–1.03),^13^ hospitalization (HR = 1.04, 95% CI: 1.02–1.07),^13^ healthcare costs, and mortality (HR = 1.05, 95% CI: 1.03–1.06)^13–16^ In LTC settings, polypharmacy is widespread; however, cognitively intact residents have been found to be prescribed significantly more medications than residents with cognitive impairment.^17^ Despite this, 82% of LTC residents receive at least one anticholinergic or sedative medication, and 26.9% receive at least one potentially inappropriate medication.^17^ These findings suggest that, despite being prescribed fewer medications overall, residents with cognitive impairment may experience a greater burden of medication-related harm because they are exposed to similarly high levels of potentially inappropriate medications.^18^ As medication regimens become increasingly complex and medication burden begins to represent potential risks in addition to expected benefits, determining which medications remain appropriate becomes increasingly challenging, particularly at the end of life.^19^

Several studies have examined medication use during the last year of life among people with dementia to better understand prescribing during this clinically complex period.^20–24^ In a UK primary care setting, people with dementia were prescribed a mean of 4.8 medications 1 year prior to death, increasing to 5.6 medications 2 months prior death and then decreasing to 4.9 at death.^21^ Cardiovascular medications were the most commonly dispensed, whereas prescriptions for gastrointestinal and central nervous system medications increased throughout the year, particularly those for laxatives, analgesics, antidepressants, and antipsychotics. The number of medications prescribed decreased with age and increased with additional comorbidities.^21^ It remains unclear how these prescribing patterns compare with those among older adults without dementia or among people with dementia residing in LTC at the end of life.

There have been no consensus guidelines developed to support clinical decision-making on reducing medication burden (polypharmacy), risk of drug interactions, or risk of adverse drug events at the end of life. Furthermore, we do not know if there are differences in medication use at the end of life according to cognitive status or place of residence. Therefore, we aimed to determine whether polypharmacy or hyperpolypharmacy persists among people with dementia throughout the final year of life and whether patterns of medication use differ between community-dwelling older adults with dementia, community-dwelling older adults without dementia, and older adults with dementia living in LTC. Our specific objectives were to examine (a) the number of medications used by people with dementia in the community compared with community-dwelling older adults without dementia or people with dementia residing in LTC during the early, mid, and late periods of the final year of life; (b) the most prescribed medications during the last year of life in these populations; and (c) sex differences in medication use among the three groups studied.

## Methods

This was a retrospective, matched case-control study of older adults with a recorded date of death between 1 January 2019 and 1 January 2021, in the province of Nova Scotia, Canada. The population of Nova Scotia is approximately 1 million, with approximately 22.2% aged 65 years or older.^25^ We compared polypharmacy, hyperpolypharmacy, and medications used among community-dwelling older adults with dementia, community-dwelling older adults without dementia, and people with dementia residing in LTC.

Administrative health data from six sources were linked by a data custodian (Health Data Nova Scotia): (a) the Insured Patient Registry (MASTER), (b) Vital Statistics (VITAL), (c) Seniors’ Pharmacare (PHARM), (d) the Canadian Institute for Health Information (CIHI) National Ambulatory Care Reporting System (NACRS), (e) Eligibility Group (EGROUP), and (f) the CIHI—Discharge Abstract Database (DAD). MASTER contains demographic information such as administrative sex and date of birth. VITAL contains information on underlying and contributing causes of death, place of birth, place of residence, age, administrative sex, marital status, hospital, and autopsy details, if applicable. PHARM includes drug and prescription information for items billed to the Seniors’ Pharmacare insurance plan. Seniors’ Pharmacare is a voluntary medication insurance program available to all Nova Scotians aged 65 years or older. Seniors’ Pharmacare covers a wide range of medications and medical products dispensed by community pharmacies. Annual enrollment in Seniors’ Pharmacare varies but includes approximately 65% of adults aged 65 years or older in the province.^26^ Medications dispensed to hospitalized patients were not included. NACRS contains details of ambulatory care visits billed in the province, whereas the DAD contains details of service transfers during hospitalization, specialty services received, and case complexity for hospitalizations. The EGROUP database contains a subset of the MASTER dataset that includes the date on which individuals become eligible to move to an LTC home.

Linked health administrative data were used to identify community-dwelling older adults with dementia. From VITAL, we identified older adults with a recorded date of death between 1 January 2019 and 1 January 2021. We then selected the last year of life as the observation period and a 1-year look-back period preceding the observation period. During this look-back period, we identified individuals with International Classification of Disease (ICD)-9 (ICD-9) and ICD-10 codes for dementia recorded in the MASTER, NACRS, and DAD datasets and then applied the administrative data definition of dementia specified by the Nova Scotia Dementia Strategy.^27^ Dementia was assigned based on an ICD-10 code for dementia having been recorded on at least three separate occasions (F01.x, F05.1, F10.6, F10.7, F04.x, F00.x, F02.x, G30.x, G31.0, G31.1, G31.8, G31.9, G32.8, G91.0, G91.2-G91.3, G91.8, G91.9, G94.x, and R54.x) within the DAD, NACRS, and MED datasets. We recorded the date of the third occasion with a dementia ICD code as the date of dementia diagnosis. We coded prescriptions dispensed on or after the date of dementia diagnosis as having been dispensed in the presence of dementia. As a final step to verify community-dwelling status at the end of life, the LTC flag in the EGROUP dataset was checked, and only individuals identified as living in the community at the time of death were included in the community-dwelling group.

Community-dwelling people without dementia were selected from Nova Scotians aged 65 years or older at the time of death who had a recorded date of death between 1 January 2019 and 1 January 2021, in the VITAL database and did not meet the dementia case definition. Community-dwelling people without dementia were matched with replacement to community-dwelling older adults with dementia based on age, administrative sex, and date of death (±30 days). We elected to allow for matching with replacement due to concerns that we would be unable to find community-dwelling older adults without dementia and LTC-dwelling older adults with dementia as matches for the controls. Allowing matching with replacement ensured that all eligible community-dwelling individuals with dementia could be matched to a comparable control while maintaining the planned matching strategy. A second comparator group (LTC dwelling people with dementia) comprised LTC dwelling people with dementia in the last year of life. This group was identified from those identified to have dementia but were identified to have the LTC flag in the EGROUP dataset at baseline (the first point of data collection) and at the time of death. This group was also matched on the same variables to community-dwelling older adults with dementia (age, administrative sex, and date of death ±30 days), again with replacement.

### Exposures and outcomes

For each individual in the cohort, we collected administrative sex, residence in an urban or rural location, Charlson Comorbidity Index, and the comorbidities hypertension, osteoarthritis, anxiety, chronic obstructive pulmonary disease, ischemic heart disease, cancer, and diabetes at study entry and on the date of death. All medication-dispensing records from the last year of life were collected.

Polypharmacy was the primary outcome and was defined as five or more prescription medications dispensed concurrently for at least 30 consecutive days. Hyperpolypharmacy was defined as 10 or more prescription medications dispensed concurrently for at least 30 consecutive days.

The prescription medication-dispensing data collected included the date on which a prescription was dispensed and the number of days for which the medication was supplied. Using these data, we created a longitudinal medication exposure dataset for each participant that included each day of follow-up during the final year of life and indicated whether each dispensed medication was considered available on that date based on the dispensing date and days supplied. If five or more medications were indicated as available for 30 consecutive days, this was counted as an episode of polypharmacy. If 10 or more medications were indicated as available for 30 consecutive days, this was counted as an episode of hyperpolypharmacy. Short courses of antibiotics therefore did not contribute to polypharmacy. Because administrative dispensing data did not identify medications prescribed on an as-needed basis, these medications were considered available during the period covered by the dispensing record if the days supplied indicated availability for more than 30 days. These definitions reflect medication availability based on dispensing data and do not confirm actual medication ingestion. We examined three distinct time periods in the year preceding death by dividing the year evenly into three periods (early, mid, and late). We identified polypharmacy and hyperpolypharmacy at cohort entry or during the early period (12–9 months prior to death), the mid-year period (8–5 months prior to death), and the final 4 months or late period (4–0 months prior to death). The most common prescription items dispensed overall and those most dispensed during each time period were reported.

### Statistical analysis

All patient details were sufficiently deidentified such that individual participants could not be identified. To further support comparisons in the descriptive analyses, we performed tests of independence. Statistical significance was set at p < 0.05, and analyses were conducted using RStudio (version 2024.09.1 + 394). During the early time period (9–12 months before death) and the late time period (0–3 months before death), we compared community-dwelling older adults with dementia with community-dwelling older adults without dementia and people with dementia residing in LTC using t-tests for continuous variables and chi-square tests for categorical variables. We compared community-dwelling older adults with dementia with community-dwelling older adults without dementia and, separately, with people with dementia residing in LTC using multivariable logistic regression models to estimate the probability of polypharmacy and hyperpolypharmacy. Models were adjusted for age, administrative sex, case/comparator status, rural or urban residence, and Charlson Comorbidity Index. Separate multivariable logistic regression models were fitted for each of the three assessed periods (early, mid, and late periods of the final year of life), with each participant contributing a single observation to each model. Although age and administrative sex were included as matching variables, they were retained a priori as covariates in all regression models because of their well-established clinical associations with medication use and end of life care. Including these variables in the adjusted analyses accounted for any residual differences remaining after matching and improved the precision of the estimated associations. The purpose of matching was to enhance comparability among the study groups rather than to eliminate the need for statistical adjustment. The most dispensed prescription medications were reported by anatomical therapeutic chemical (ATC) code for the overall final year of life and for each time period. Sex-stratified analyses were also conducted to examine sex-based differences in polypharmacy and hyperpolypharmacy. The reporting of this study conforms to the Strengthening the Reporting of Observational Studies in Epidemiology (STROBE) guidelines.^28^

### Ethical approval

This study was conducted in accordance with the Declaration of Helsinki of 1975, as revised in 2024. This study received ethics approval from the Nova Scotia Health Research Ethics Board (REB File No. 1027635), with subsequent renewals. The study was also approved by the Health Data Nova Scotia Data Access Committee.

## Results

We identified 1274 community-dwelling older adults with dementia; each was matched with replacement to a community-dwelling person without dementia and a LTC dwelling person with dementia. Table 1 shows demographic information for the 3706 unique participants across community-dwelling older adults with dementia, community-dwelling older adults without dementia, and LTC dwelling people with dementia at time of cohort entry and death. Only 48 community-dwelling older adults without dementia were resampled and 68 individuals in LTC with dementia were resampled, representing just 3% of matched controls and suggesting that repeated use of controls was uncommon. Community-dwelling people with dementia were more likely to have recorded diagnoses of osteoarthritis, anxiety, or ischemic heart disease 1 year before death (p < 0.02) than the comparators. Community-dwelling people without dementia were more likely than the other groups to have hypertension or a cancer diagnosis during the final year of life (p < 0.001). People with dementia residing in LTC had lower Charlson Comorbidity Index scores than community-dwelling older adults with dementia or community-dwelling older adults without dementia during the early and late time periods.

**Table 1.** Characteristics of PLWD cases, community controls, PLWD LTC at baseline (one year before death) and at the date of death.

	Overall (%)	PLWD cases	Community controls	PLWD LTC	p value^a^
n	3706.0	1274.0	1226.0	1206.0	
Mean age (SD)	84.80 (7.95)	84.83 (7.98)	84.64 (7.96)	84.94 (7.93)	0.637
Sex = male (%)	1482 (40.0)	512 (40.2)	497 (40.5)	473 (39.2)	0.79
Urban residence (%)	2343 (63.2)	824 (64.7)	739 (60.3)	780 (64.7)	0.033
One year before death					
Hypertension (%)	1686 (45.5)	652 (51.2)	638 (52.0)	396 (32.8)	<0.001
Osteoarthritis (%)	537 (14.5)	211 (16.6)	154 (12.6)	172 (14.3)	0.017
Anxiety (%)	698 (18.8)	285 (22.4)	156 (12.7)	257 (21.3)	<0.001
COPD (%)	1033 (27.9)	348 (27.3)	370 (30.2)	315 (26.1)	0.071
Ischemic heart disease (%)	851 (23.0)	366 (28.7)	294 (24.0)	191 (15.8)	<0.001
Cancer (%)	581 (15.7)	210 (16.5)	265 (21.6)	106 (8.8)	<0.001
Diabetes (%)	1024 (27.6)	365 (28.6)	345 (28.1)	314 (26.0)	0.308
Charlson Comorbidity Index 0–1 (%)	761 (20.5)	63 (4.9)	403 (32.9)	295 (24.5)	<0.001
Charlson Comorbidity Index 2–3 (%)	1783 (48.1)	667 (52.4)	463 (37.8)	653 (54.1)	
Charlson Comorbidity Index 4–5 (%)	671 (18.1)	295 (23.2)	198 (16.2)	178 (14.8)	
Charlson Comorbidity Index 6+ (%)	491 (13.2)	249 (19.5)	162 (13.2)	80 (6.6)	
At time of death					
Hypertension (%)	1331 (35.9)	498 (39.1)	586 (47.8)	247 (20.5)	<0.001
Osteoarthritis (%)	490 (13.2)	175 (13.7)	134 (10.9)	181 (15.0)	0.01
Anxiety (%)	721 (19.5)	255 (20.0)	179 (14.6)	287 (23.8)	<0.001
COPD (%)	1442 (38.9)	474 (37.2)	519 (42.3)	449 (37.2)	0.011
Ischemic heart disease (%)	1194 (32.2)	438 (34.4)	500 (40.8)	256 (21.2)	<0.001
Cancer (%)	939 (25.3)	324 (25.4)	502 (40.9)	113 (9.4)	<0.001
Diabetes (%)	980 (26.4)	329 (25.8)	351 (28.6)	300 (24.9)	0.091
Charlson Comorbidity Index 0–1 (%)	326 (8.8)	78 (6.1)	129 (10.5)	119 (9.9)	<0.001
Charlson Comorbidity Index 2–3 (%)	1545 (41.7)	481 (37.8)	344 (28.1)	720 (59.7)	
Charlson Comorbidity Index 4–5 (%)	824 (22.2)	330 (25.9)	271 (22.1)	223 (18.5)	
Charlson Comorbidity Index 6+ (%)	1011 (27.3)	385 (30.2)	482 (39.3)	144 (11.9)	

LTC: long-term care; PLWD: people living with dementia; COPD: chronic obstructive pulmonary disease.

a Significant p values demonstrate that at least one group is different from the others.

### Polypharmacy and hyperpolypharmacy

The overall prevalence of polypharmacy at any time during the last year of life was 50.9% (1944/3822) and the overall prevalence of hyperpolypharmacy at any time in the last year of life was 8.69% (332/3822). We show the proportions by time period (early, mid, and late) and case/comparator group in Figure 1. Our descriptive analysis showed that community-dwelling older adults with dementia had the highest rates of polypharmacy and hyperpolypharmacy across all time periods assessed. The odds of polypharmacy during the early time period were lower among community-dwelling older adults without dementia than among community-dwelling older adults with dementia (adjusted odds ratio (aOR) = 0.83, 95% CI: 0.70–0.99, p = 0.036). In models of the entire group, a Charlson Comorbidity Index greater than zero at baseline was associated with increased odds of polypharmacy overall and during the mid-year period (see Figure 2). Similarly, a Charlson Comorbidity Index greater than zero was consistently associated with increased odds of hyperpolypharmacy at all time points (see Figure 2). In the entire group, increasing age was associated with hyperpolypharmacy at all time points (overall aOR = 0.97, 95% CI: 0.96–0.99, p < 0.0014).

**Figure 1.**
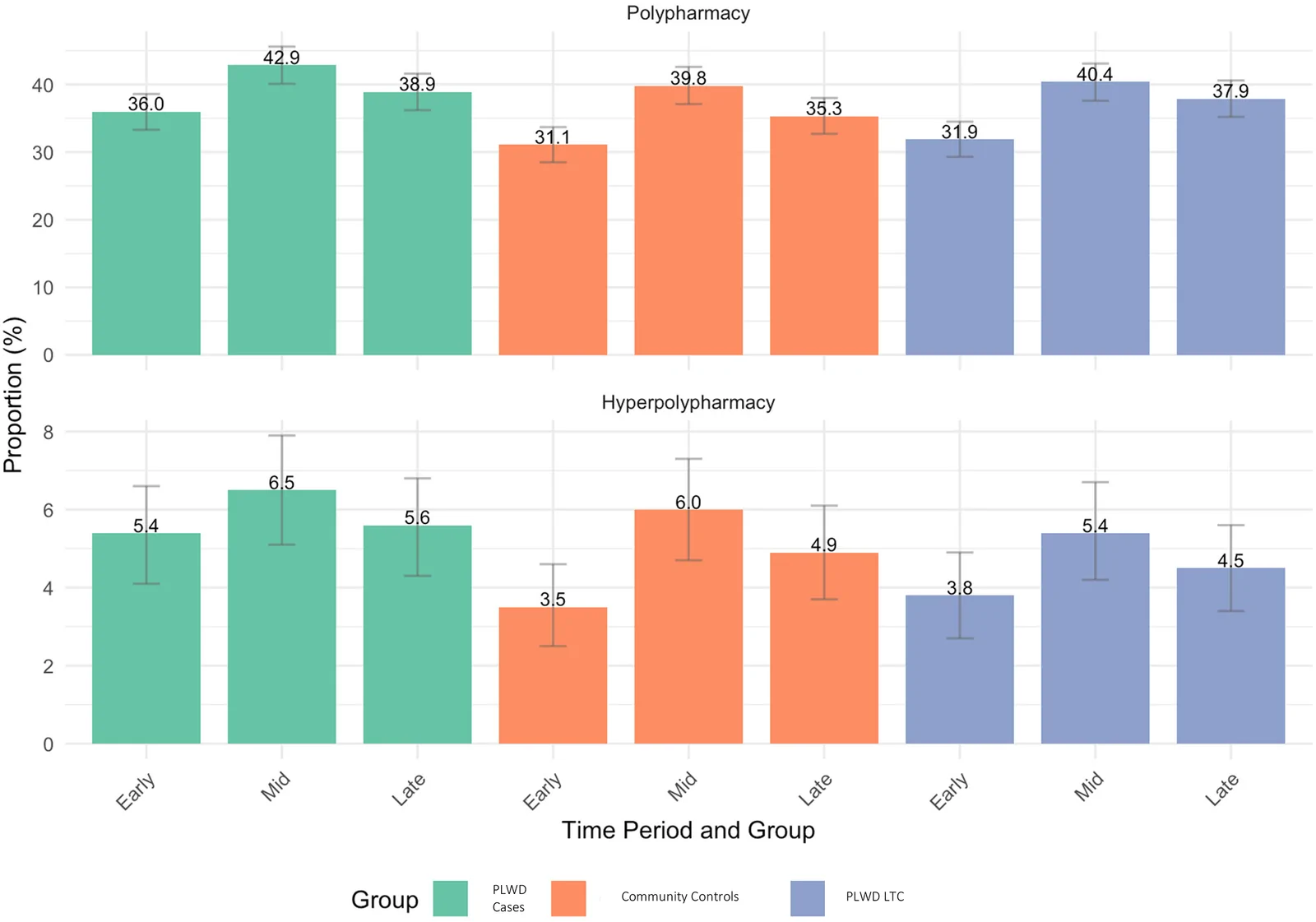
Proportions of polypharmacy and hyperpolypharmacy in PLWD cases, community controls, and PLWD LTC in early period (12–9 months prior to death), the mid-period (8–5 months prior to death), and the late period (4–0 months prior to death).

LTC: long-term care; PLWD: people living with dementia.

**Figure 2.**
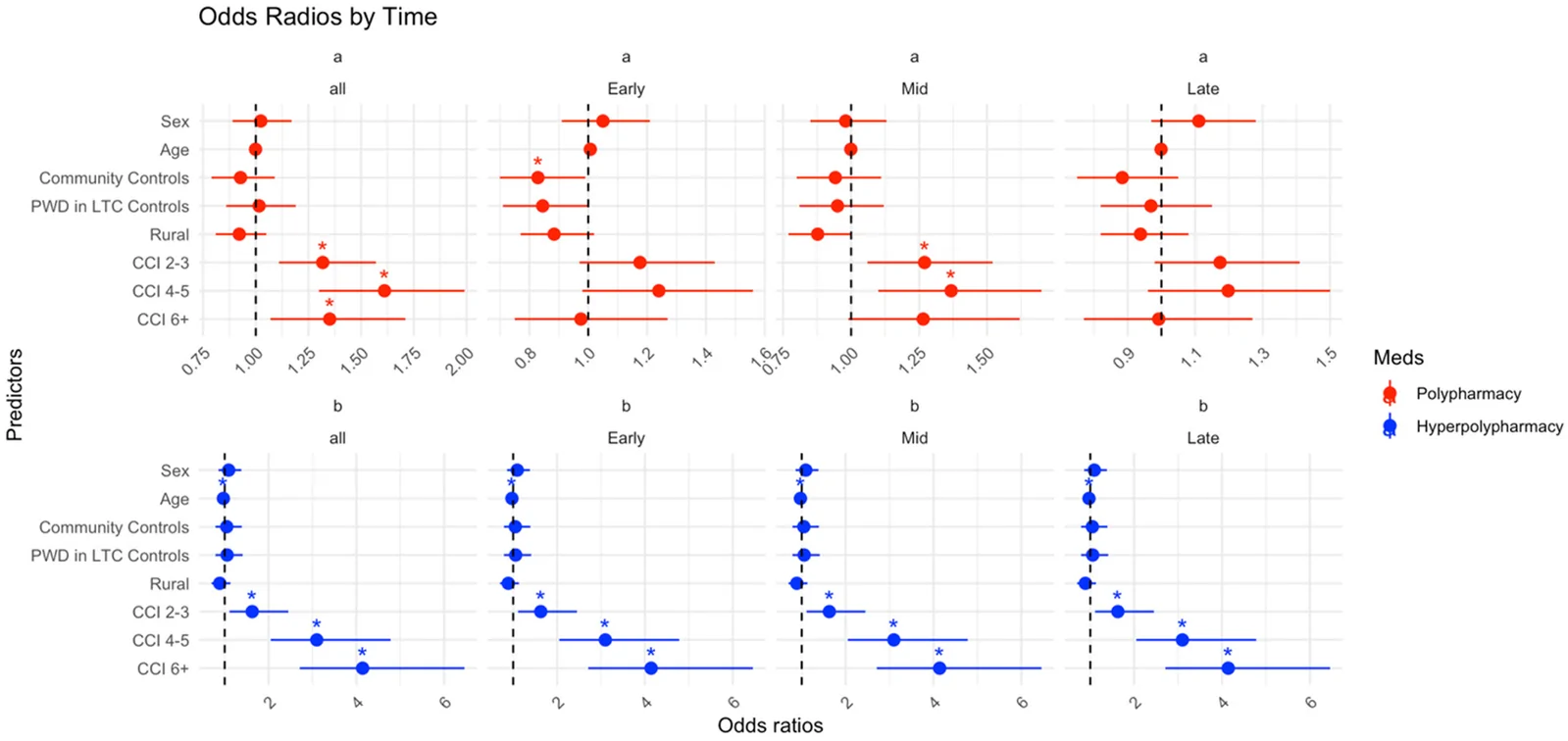
Adjusted odds ratio for risk of polypharmacy and hyperpolypharmacy in PLWD cases, community controls, and PLWD LTC overall, in early period (12–9 months prior to death), the mid-period (8–5 months prior to death), and the late period (4–0 months prior to death).

LTC: long-term care; PLWD: people living with dementia.

### Medications

The 12 most commonly prescribed items at the end of life are listed in the Appendix and shown by time period in the heat map in Figure 3. Furosemide, levothyroxine, and salbutamol were the most prevalent medications during the last year of life. Trends in medications dispensed are displayed in the heat map. For example, rosuvastatin, atorvastatin, and citalopram were more frequently present during the early period but were absent as the end of life approached. Hydromorphone emerged in the last 4 months of life as a prevalent medication.

**Figure 3.**
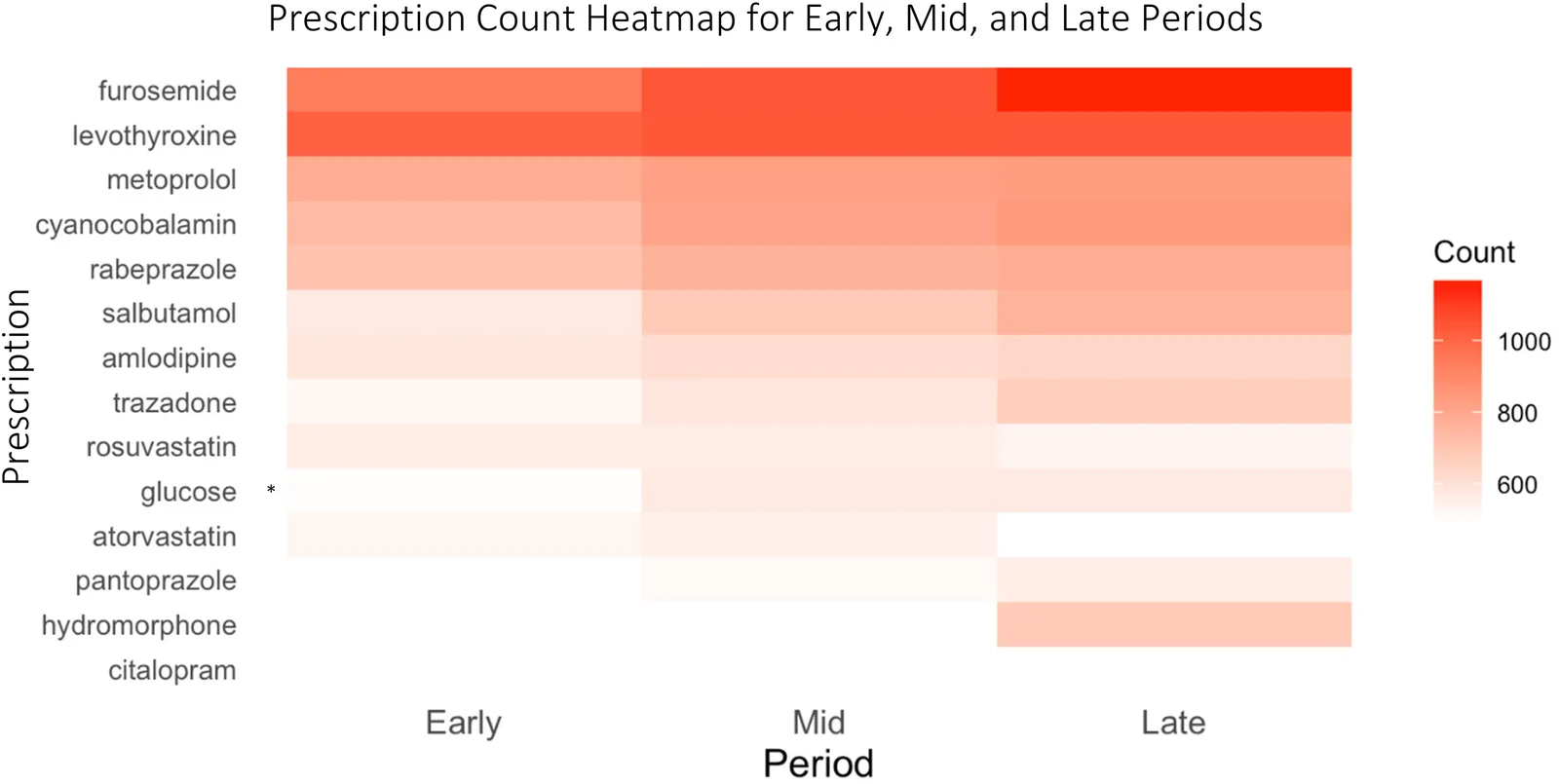
Heat map to demonstrate how the 12 most common prescriptions by patient count and time period (overall and in the early period (12–9 months prior to death), the mid-period (8–5 months prior to death), and the late period (4–0 months prior to death)) change in frequency of dispensation over the last year of life.

*glucose = glucose testing strips.

### Sex-stratified analyses

Known sex differences in health behaviors^29,30^ and differences in prescribing to men and women,^31–33^ combined with sex differences^34^ in disease prevalence^35–37^ prompted a sex-stratified analysis. Those with an administrative sex recorded as female were older and less likely to have comorbidities. Participants with an administrative sex recorded as male were more likely to experience polypharmacy and hyperpolypharmacy during each time period. Evaluating by time period, polypharmacy among females ranged from a low of 31.1% in the early time point among LTC dwelling people with dementia to a high of 42.3% in the midpoint among community-dwelling older adults with dementia. For males, the lowest prevalence of polypharmacy was 29.9% in the early period among community-dwelling older adults without dementia, and the highest was 43.7% at the midpoint among community-dwelling older adults with dementia. For both males and females, those with higher Charlson Comorbidity Indexes had increased odds of polypharmacy and hyperpolypharmacy. For females, rurality was associated with an increased odds of polypharmacy in the overall time period and specifically in the early and mid-time periods (see Figure 4 and the Appendix for details).

**Figure 4.**
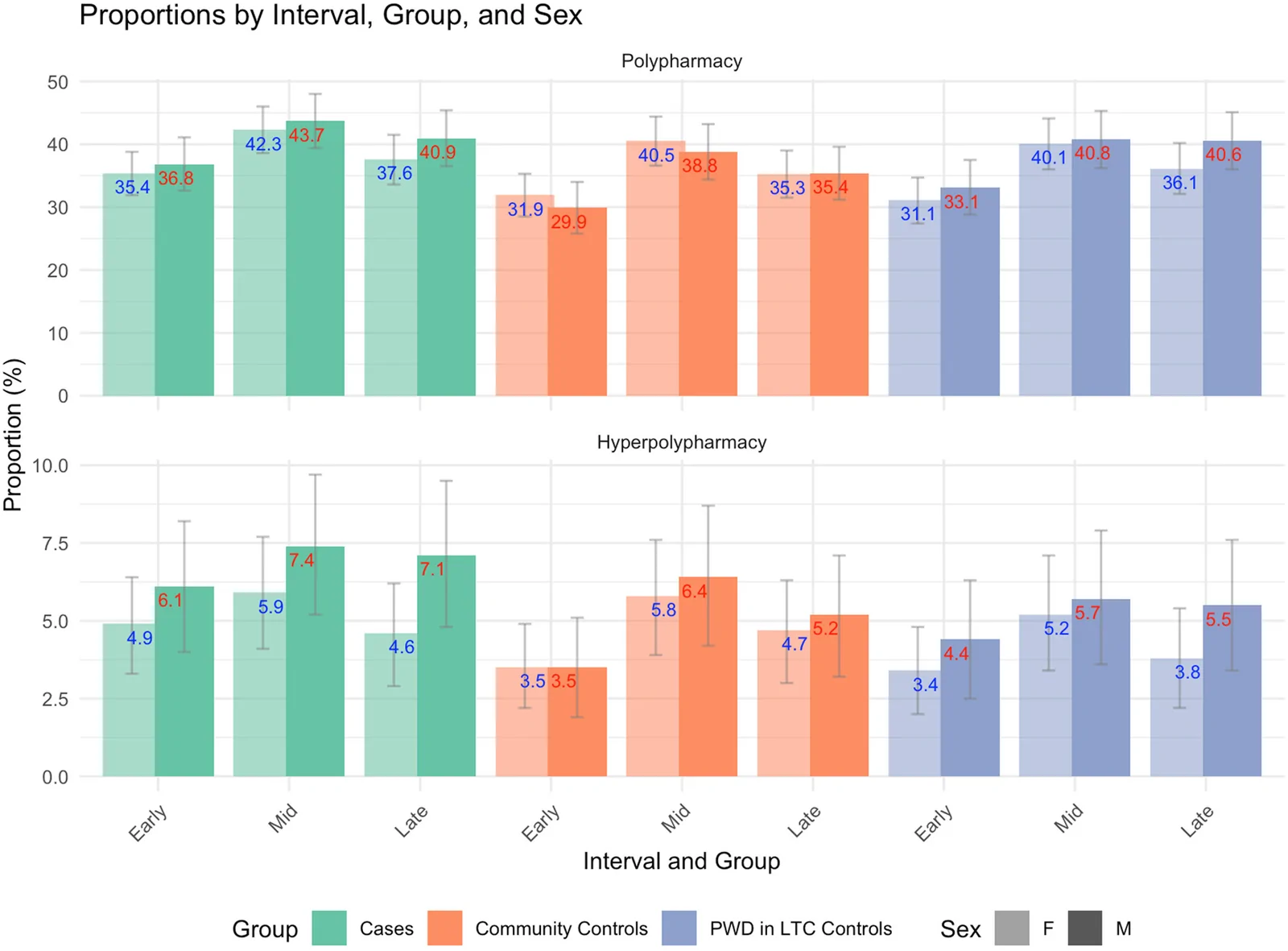
Sex-Stratified proportions of polypharmacy and hyperpolypharmacy in PLWD Cases, community controls, and PLWD LTC in early period (12–9 months prior to death), the mid-period (8–5 months prior to death), and the late period (4–0 months prior to death).

LTC: long-term care; PLWD: people living with dementia.

## Discussion

We identified that, within the last year of life, more than half of community-dwelling older adults with dementia, community-dwelling older adults without dementia, and older adults with dementia residing in LTC had experienced polypharmacy, and approximately one-tenth had experienced hyperpolypharmacy. Community-dwelling older adults with dementia had the highest rates of polypharmacy and hyperpolypharmacy across all time periods assessed. This finding supports that polypharmacy is more prevalent in older adults living with dementia in the last year of life compared with those without dementia in the community. Perhaps surprisingly, older adults living with dementia in LTC had lower prevalence of polypharmacy compared with community-dwelling older adults with dementia. The odds of polypharmacy during the early time period were lower among community-dwelling older adults without dementia than among community-dwelling older adults with dementia. Considering the potential factors associated with hyperpolypharmacy, a Charlson Comorbidity Index greater than zero was consistently associated with higher odds of hyperpolypharmacy at all time points among both those with an administrative sex of male and female. Females were older and less likely to be living with comorbidities compared to males, and this seems to correspond with the finding that males were more likely to experience polypharmacy and hyperpolypharmacy during each time period.

The high rates of polypharmacy identified in all three groups during the last year of life are consistent with rates previously reported among older adults in Canada. CIHI reports in 2021 identified that 47.6% of Canadian seniors were on five or more medications chronically and 11.6% of Canadian seniors were consistently on 10 or more medications.^38^ We demonstrated a slightly higher rate of polypharmacy (50.9%) and slightly lower rate of hyperpolypharmacy (8.7%) in this limited Nova Scotian population of adults over 65 years of age in the last year of life. The CIHI report focused on all older adults aged 65 years of age and older and not just those at the end of life so considering both studies and both populations have similar findings suggests that the counts of medications used across the senior years do not change as drastically as we may expect. For policymakers and healthcare planners, our findings highlight that a high medication burden persists among older adults with dementia approaching the end of life. However, comparisons with CIHI estimates should be interpreted with caution because those estimates include older adults who survived the observation period, whereas our study specifically examined decedents. Similar levels of medication burden across these populations suggest an ongoing need for medication optimization but do not establish that end of life prescribing patterns are indistinguishable from those earlier in the disease trajectory. In other populations near end of life there are some differences. In a Jordanian population of hospitalized older adults with planned to transition to hospice care polypharmacy rates were higher than our population at 63.0%.^39^ A global assessment of polypharmacy among older adults with dementia reported a pooled prevalence estimate of 62% (95% CI: 52–71), which exceeds the rate we identified.^40^ It is possible that deprescribing efforts had been initiated prior to the final year of life and is reflected in our rate of polypharmacy that is lower than the global average.

In comparison with other jurisdictions, a previous study by Denholm et al.^21^ examined prescribing among people with dementia with a mean age of 86.6 years in UK primary care practices during the final year of life. Similar to our results, the mid-period of the final year of life was found to have the highest amount of medication use. Our finding that polypharmacy and hyperpolypharmacy were highest during the midpoint of the last year of life may be related to access to prescriber care. Older adults in the mid-period of their last year of life, particularly those who are community-dwelling, may still have the ability to mobilize independently, making primary care provider visits possible. Reduced ability to walk is recognized as being associated with an increased risk of mortality.^41^ A primary care provider visit provides opportunities for prescribing (and deprescribing) so when mobility declines during the final 3 months of life, access to primary care and deprescribing interventions may be less possible.^42^ It is also possible that the midpoint of the last year of life represents a time when the perceived health status of the individual is recognized as changing and a trial of increasing intervention is appropriate in an attempt to address potentially reversible health problems to counter a growing burden of frailty.^43^ This may be compounded by a common frailty trajectory among those approaching the end of life—an initial increase followed by a plateau,^43^ which is challenging for prognostication and the subsequent rationalizing of medications.

Although changes in medications dispensed across the three time periods varied only slightly, the findings are consistent with other jurisdictions.^44^ If one considers specifically those receiving hospice care, a UK study of six hospices with a patient population aged 56–85 years showed that polypharmacy varied by site from a low of 41.7%–90.9%. In the study of hospice populations in the UK, older age was actually associated with a lower risk of polypharmacy.^45^ In a study by Blass et al.,^46^ medication use among nursing home residents with advanced dementia were examined to demonstrate that at follow-up that medications for gastrointestinal complaints, opioids, and pulmonary conditions were more common closer to the end of life and that drugs used to treat mood disorders, cardiovascular drugs, antibiotics, antidementia drugs, and nonopioid pain medications were less common. Our findings showed that proton pump inhibitors and salbutamol were among the most frequently dispensed during the final year of life and that opioids, specifically hydromorphone, appear in the top ten drugs only during the 4 months immediately prior to death. We also observed a decrease in the dispensing of antidepressants (citalopram) over the last year of life. Beta-blockers remained in the top five medications dispensed across all time periods. Our findings are consistent with previous investigations of beta-blocker use among nursing home residents, which showed that approximately half of nursing home residents were prescribed a beta-blocker in the last year of life; these authors also followed residents over the last year of life and identified that 68.6% remained on a beta-blocker during the final 45 days of life.^47^

Although prior reports have suggested that antidepressants are less frequently dispensed during the final months of life,^46^ we found that trazodone remained among the most frequently dispensed medications across all three time periods. Trazodone is classified as an antidepressant but may be used for its sedative properties^48,49^ or to treat behavioral or psychological symptoms of dementia, although the most recent Canadian guidelines do not recommend it for this indication.^50^ Among older adults living with or without dementia, particularly those who are frail, the risks associated with trazodone include an increased incidence of syncope and falls (58.3% vs. 21.2%, p = 0.001)^51^ with trazodone use predicting syncope and falls independently of age, disability, and previous fall history.^51^ This prevalent use of trazodone may represent inappropriate prescribing and a possible target for deprescribing interventions. However, this is complicated by the lack of a deprescribing guideline for trazodone. There are deprescribing guidelines and algorithms available for benzodiazepines and other sedatives (Z-drugs).^52^ Anxiety and depressive disorders are among the most common mental disorders globally and represent a substantial burden among older adults.^53^ Consequently, continued trazodone use may represent a missed opportunity to optimize treatment when insomnia occurs secondary to an underlying mood or anxiety disorder. In these circumstances, treatment of the underlying condition with evidence-based therapies may improve both mood symptoms and sleep while avoiding the additional sedative burden associated with trazodone.

There were differences in the prevalence of polypharmacy and hyperpolypharmacy for males compared to females. Males had a higher frequency of polypharmacy across all time periods. This differs from some previous investigations suggesting that women experience more polypharmacy.^54,55^ We found that, among both males and females, greater comorbidity was associated with increased odds of polypharmacy, consistent with previous studies.^56^ We hypothesize that in our case-control study the population of males included lived with higher rates of comorbidity, as evidenced by the Charlson comorbidity scores, and were therefore prescribed more medications. The observed differences in polypharmacy among females who resided in a rural location compared with those residing in an urban setting highlight the potential influence of geographic inequities on medication management. Although the mechanisms underlying these differences cannot be determined from administrative data, variation in access to primary care, geriatric services, pharmacist-led medication reviews, and other medication optimization supports may contribute. Future research should explore whether targeted approaches, including telehealth-enabled medication reviews or outreach-based pharmacy services, improve equitable access to deprescribing and medication optimization for older adults living in rural communities.

It is also possible that there were some fundamental differences among the three groups studied. For example, community-dwelling older adults without dementia were more likely to live with cancer during the last year of life and likely were able to remain at home until death (or very close to the end of life) rather than those living with dementia who were less likely to live with cancer. Because community-dwelling older adults with dementia did not have institutional support, they may have been assessed as requiring polypharmacy, especially purposeful prescribed psychotropic polypharmacy,^57^ for the continued management of behavioral and psychological symptoms of dementia in the community. It is possible that, even at a time when relocation to LTC was preferred, placement may not have been available because of long LTC wait lists and other access barriers. A patient management strategy of psychotropic polypharmacy may have been selected in an effort to manage community-dwelling older adults with dementia's behavioural and psychological symptoms of dementia even with the known risks of emergency department visit or hospitalization that exist as medication burden increases.^58^ The challenges of the last year of life should not be understated and must be considered within the context and complexity of a dementia diagnosis, as was present in our population. We also identified higher levels of cardiovascular medications being prescribed to males in the study population. Unexpectedly, females in rural locations were more likely to experience polypharmacy than females in urban locations. This finding may be related to loneliness and social isolation in rural settings, which may be more commonly experienced by women and are known to be associated with polypharmacy.^59^ It may also reflect differences in access to primary care and opportunities for deprescribing.

Deprescribing continues to be difficult to incorporate into practice, even for older adults at the end of life who are prescribed medications for which evidence supports deprescribing. We anticipated observing deprescribing of statin medications, vitamin B12, and glucose-testing strips. This was not the case for vitamin B12 or glucose-testing strips, and while we witnessed some decline in statin use across the periods of the last year of life, we expected a more marked decrease. Statin deprescribing at the end of life is supported by evidence-based deprescribing guidelines and algorithms.^52,60,61^ Evidence suggests that vitamin B12 is commonly prescribed inappropriately^62^ and that this use often persists among older adults. Another consideration is that vitamin B12 prescription may have been related to the concern of deficiency associated with long-term proton pump inhibitor use,^63–65^ which only raises further questions about opportunities for proton pump inhibitor deprescribing.^66^ We also noted that glucose-testing strips were among the most commonly dispensed items during the last year of life. Clinical guidelines for glycemic control in frail older adults recommend more relaxed glycemic targets and reduced use of medications that induce hypoglycemia. Given that two-thirds of people were living with dementia, so it is surprising that glucose-testing strips were continued to be used frequently, even during the last few months of life.

The finding that people with dementia residing in LTC had lower Charlson comorbidity scores than both community-dwelling older adults with dementia and community-dwelling older adults without dementia was unexpected. Although one might expect LTC residents to have greater medical complexity and frailty than age-matched community-dwelling older adults, our findings suggest that factors other than medical comorbidity may contribute substantially to LTC placement among people with dementia. Cognitive impairment, functional decline, neuropsychiatric symptoms, and associated caregiver burden may represent important drivers of placement, even among individuals with fewer medical comorbidities. Further research comparing pathways to LTC among older adults with and without dementia is needed to better understand how clinical needs, caregiver factors, and system-level factors influence placement decisions.

Administrative medication data do not capture patient preferences, degree of dementia, level of functional impairment, level of behavioral or psychological symptoms, goals of care, clinician decision-making, or the clinical rationale underlying prescribing and deprescribing decisions. Therefore, the observed medication patterns cannot distinguish potentially inappropriate medication use from situations in which continuation reflected individualized treatment goals or patient preferences. Limitations of this study included those inherent in the use of administrative data such as time considerations (e.g. the specific year chosen for the cohort and controls) and location (the province of Nova Scotia). We could not account for regional prescribing patterns or changes in the healthcare workforce. We also acknowledge that the repeated use of matched controls may have affected the independence of observations. Prescription dispensing data from the Nova Scotia Seniors’ Pharmacare Program do not capture older adults who receive medication coverage from other sources or who do not participate in the program. Individuals who are not enrolled may differ from Pharmacare beneficiaries in important ways, including socioeconomic status, access to private drug coverage, and employment-related benefit history (e.g. federal government or military benefits). Because medication use, healthcare access, and dementia-related care needs may differ across these groups, selection into the Pharmacare program may limit the generalizability of our findings to all older adults in Nova Scotia. However, the direction and magnitude of any potential selection effects are uncertain.

Our findings suggest there is a potential opportunity to use policy or interventions to support deprescribing at the end of life. Focused implementation of deprescribing guidelines when patients exhibit signals of limited life expectancy may be considered. We acknowledge that we had the advantage of the retrospective nature of the study identifying when death occurred. In practice, the end of life may be a total surprise for prescribers or may have been anticipated. We recognize the difficult decisions prescribers must make with limited information, which may make the time to initiate deprescribing at the end of life difficult to identify. In our current Canadian climate with limited access to primary care providers, collaboration with allied healthcare professionals may provide opportunities such as positioning/empowering pharmacists or nurses to carry out algorithm-supported deprescribing. Evidence supporting improvements in medication appropriateness through pharmacist-led medication review is robust, and evidence syntheses suggest that pharmacists are well positioned to improve medication appropriateness among older adults.^67,68^ Many medications that may be appropriate for deprescribing are already addressed by evidence-based deprescribing guidelines and algorithms that could be readily implemented in clinical practice.^52^ These include algorithms for proton pump inhibitor discontinuation,^69^ statin deprescribing,^70^ deintensification of glucose-lowering therapy,^71^ and reduction of unnecessary blood glucose monitoring,^72^ as well as the STOPP-Frail criteria,^73^ which provide recommendations for optimizing medication use in frail older adults. Resourcing pharmacists or nurses to lead algorithm-supported deprescribing initiatives, particularly in LTC settings, could provide a practical approach to optimizing medication use among frail older adults.

## Conclusions

Polypharmacy and hyperpolypharmacy were common at the end of life, with little change observed throughout the last year of life. These findings suggests that there are opportunities for supported deprescribing in these populations, with potential benefits for older adults’ health, healthcare, and quality of life.

## Supplemental Material

sj-docx-1-imr-10.1177_03000605261487270 - Supplemental material for Polypharmacy and patterns of medication use in the final year of life among people living with dementia in Canada: A case-control study Supplemental material, sj-docx-1-imr-10.1177_03000605261487270 for Polypharmacy and patterns of medication use in the final year of life among people living with dementia in Canada: A case-control study by Jasmine Mah, Catherine Richer, Abby Emdin, Kenneth Rockwood, Samuel Stewart, Melissa K Andrew and Shanna C Trenaman in Journal of International Medical Research

sj-doc-2-imr-10.1177_03000605261487270 - Supplemental material for Polypharmacy and patterns of medication use in the final year of life among people living with dementia in Canada: A case-control study Supplemental material, sj-doc-2-imr-10.1177_03000605261487270 for Polypharmacy and patterns of medication use in the final year of life among people living with dementia in Canada: A case-control study by Jasmine Mah, Catherine Richer, Abby Emdin, Kenneth Rockwood, Samuel Stewart, Melissa K Andrew and Shanna C Trenaman in Journal of International Medical Research

## Data Availability

The datasets used and/or analyzed during the current study are available from the corresponding author on reasonable request, subject to completion of REB and data custodian privacy training and approval of data access.
