# Supplementary material for: Polypharmacy and patterns of medication use in the final year of life among people living with dementia in Canada: A case-control study
Source: J Int Med Res. 2026 Sep 27;54(9):03000605261487270. doi: 10.1177/03000605261487270 (PMC13735685; doi:10.1177/03000605261487270)
Supplement: Supplementary file 1 [file sj-docx-1-imr-10.1177_03000605261487270.docx]

**Appendix 1**

*Table A1. Top twelve most* *common prescriptions by patient count overall and in the early period (12 to 9 months prior to death), the mid period (8 to 5 months prior to death), and the late period (4 to 0 months prior to death).*

| **ATC** | **Prescription** | **All** | **Early** | **Mid** | **Late** |
| --- | --- | --- | --- | --- | --- |
| C03CA01 | furosemide | 1273 | 933 | 1039 | 1167 |
| H03AA01 | levothyroxine | 1066 | 1014 | 1037 | 1039 |
| R03AC02 | salbutamol | 1035 | 573 | 681 | 761 |
| B03BA01 | cyanocobalamin | 923 | 736 | 813 | 847 |
| C07AB02 | metoprolol | 907 | 783 | 822 | 835 |
| A02BC04 | rabeprazole | 902 | 700 | 762 | 785 |
| J01DB01 | cefalexin | 792 | 266 | 349 | 435 |
| V04CA02 | glucose test strips | 760 | 503 | 571 | 570 |
| N06AX05 | trazadone | 756 | 526 | 585 | 666 |
| N02AA03 | hydromorphone | 756 | 259 | 329 | 679 |
| C08CA01 | amlodipine | 716 | 584 | 615 | 635 |
| H02AB07 | prednisone | 635 | 305 | 372 | 459 |

*Table A2:* *Sex-Stratified Characteristics of Cases, Community Controls, LTC Controls at baseline (one year before death) and at the date of death*

| Sex | Females | Males | p-value |
| --- | --- | --- | --- |
| "n" | 2224 | 1482 |  |
| Mean age (SD) | **86.42 (8.16)** | **82.38 (6.97)** | **<0.001** |
| Urban residence (%) | 1431 (64.3) | 912 (61.5) | 0.089 |
| Case or control |  |  |  |
| Case | 762 (34.3) | 512 (34.5) | 0.79 |
| Community control | 729 (32.8) | 497 (33.5) |  |
| LTC control | 733 (33.0) | 473 (31.9) |  |
| **One year Before Death** |  |  |  |
| Hypertension (%) | **1045 (47.0)** | **641 (43.3)** | **0.028** |
| Osteoarthritis (%) | **354 (15.9)** | **183 (12.3)** | **0.003** |
| Anxiety (%) | 433 (19.5) | 265 (17.9) | 0.243 |
| COPD (%) | 595 (26.8) | 438 (29.6) | 0.068 |
| Ischemic heart disease (%) | **437 (19.6)** | **414 (27.9)** | **<0.001** |
| Cancer (%) | **277 (12.5)** | **304 (20.5)** | **<0.001** |
| Diabetes (%) | **546 (24.6)** | **478 (32.3)** | **<0.001** |
| Charlson Comorbidity Index 0-1 (%) | **522 (23.5)** | **239 (16.1)** | **<0.001** |
| Charlson Comorbidity Index 2-3 (%) | **1091 (49.1)** | **692 (46.7)** |  |
| Charlson Comorbidity Index 4-5 (%) | **371 (16.7)** | **300 (20.2)** |  |
| Charlson Comorbidity Index 5+ (%) | **240 (10.8)** | **251 (16.9)** |  |
| **At Death** |  |  |  |
| Hypertension (%) | 813 (36.6) | 518 (35.0) | 0.336 |
| Osteoarthritis (%) | **335 (15.1)** | **155 (10.5)** | **<0.001** |
| Anxiety (%) | **467 (21.0)** | **254 (17.1)** | **0.004** |
| COPD (%) | **777 (34.9)** | **665 (44.9)** | **<0.001** |
| Ischemic heart disease (%) | **649 (29.2)** | **545 (36.8)** | **<0.001** |
| Cancer (%) | **480 (21.6)** | **459 (31.0)** | **<0.001** |
| Diabetes (%) | **510 (22.9)** | **470 (31.7)** | **<0.001** |
| Charlson Comorbidity Index 0-1 (%) | **224 (10.1)** | **102 (6.9)** | **<0.001** |
| Charlson Comorbidity Index 2-3 (%) | **1005 (45.2)** | **540 (36.4)** |  |
| Charlson Comorbidity Index 4-5 (%) | **471 (21.2)** | **353 (23.8)** |  |
| Charlson Comorbidity Index 5+ (%) | **524 (23.6)** | **487 (32.9)** |  |

*Table A3: Sex-Stratified regression models*

Polypharmacy analysis for females

| **Variable** | **Estimate** | **P value** |
| --- | --- | --- |
| All time periods | | |
| (Intercept) | 0.858 [0.33, 2.23] | 0.753 |
| Age | 1.000 [0.99, 1.01] | 0.926 |
| Community Controls | 1.039 [0.84, 1.29] | 0.723 |
| PLWD LTC | 0.976 [0.79, 1.20] | 0.823 |
| Rural | 0.795 [0.67, 0.94] | 0.009 |
| Charlson Comorbidity Score on entry 1-2 | 1.316 [1.06, 1.63] | 0.012 |
| Charlson Comorbidity Score on entry 3-4 | 1.511 [1.15, 1.99] | 0.003 |
| Charlson Comorbidity Score on entry 5+ | 1.542 [1.12, 2.13] | 0.008 |
| Early | | |
| (Intercept) | 0.237 [0.08, 0.67] | 0.007 |
| Age | 1.010 [1.00, 1.02] | 0.082 |
| Community Controls | 0.901 [0.72, 1.13] | 0.372 |
| PLWD LTC | 0.856 [0.68, 1.07] | 0.178 |
| Rural | 0.728 [0.61, 0.88] | 0.001 |
| Charlson Comorbidity Score on entry 1-2 | 1.192 [0.94, 1.51] | 0.147 |
| Charlson Comorbidity Score on entry 3-4 | 1.262 [0.94, 1.70] | 0.126 |
| Charlson Comorbidity Score on entry 5+ | 1.097 [0.77, 1.56] | 0.606 |
| Mid | | |
| (Intercept) | 0.799 [0.30, 2.14] | 0.656 |
| Age | 0.999 [0.99, 1.01] | 0.905 |
| Community Controls | 0.969 [0.78, 1.20] | 0.773 |
| PLWD LTC | 0.953 [0.77, 1.18] | 0.661 |
| Rural | 0.776 [0.65, 0.93] | 0.005 |
| Charlson Comorbidity Score on entry 1-2 | 1.120 [0.90, 1.40] | 0.318 |
| Charlson Comorbidity Score on entry 3-4 | 1.100 [0.83, 1.46] | 0.509 |
| Charlson Comorbidity Score on entry 5+ | 1.336 [0.96, 1.85] | 0.082 |
| Late | | |
| (Intercept) | 0.508 [0.19, 1.38] | 0.185 |
| Age | 1.002 [0.99, 1.01] | 0.737 |
| Community Controls | 0.938 [0.75, 1.17] | 0.570 |
| PLWD LTC | 0.965 [0.78, 1.20] | 0.749 |
| Rural | 0.854 [0.71, 1.02] | 0.084 |
| Charlson Comorbidity Score on entry 1-2 | 1.130 [0.90, 1.42] | 0.289 |
| Charlson Comorbidity Score on entry 3-4 | 1.092 [0.82, 1.46] | 0.548 |
| Charlson Comorbidity Score on entry 5+ | 1.147 [0.82, 1.60] | 0.424 |

Hyperpolypharmacy analysis for females

| **Variable** | **Estimate** | **P value** |
| --- | --- | --- |
| All time periods | | |
| (Intercept) | 0.621 [0.11, 3.53] | 0.59359 |
| Age | 0.970 [0.95, 0.99] | 0.00204 |
| Community Controls | 1.220 [0.83, 1.80] | 0.31388 |
| PLWD LTC | 1.135 [0.76, 1.69] | 0.5338 |
| Rural | 0.911 [0.66, 1.26] | 0.57029 |
| Charlson Comorbidity Score on entry 1-2 | 1.481 [0.94, 2.41] | 0.10201 |
| Charlson Comorbidity Score on entry 3-4 | 2.232 [1.31, 3.85] | 0.00334 |
| Charlson Comorbidity Score on entry 5+ | 3.286 [1.89, 5.77] | 0.00003 |
| Early | | |
| (Intercept) | 0.621 [0.11, 3.53] | 0.59359 |
| Age | 0.970 [0.95, 0.99] | 0.00204 |
| Community Controls | 1.220 [0.83, 1.80] | 0.31388 |
| PLWD LTC | 1.135 [0.76, 1.69] | 0.5338 |
| Rural | 0.911 [0.66, 1.26] | 0.57029 |
| Charlson Comorbidity Score on entry 1-2 | 1.481 [0.94, 2.41] | 0.10201 |
| Charlson Comorbidity Score on entry 3-4 | 2.232 [1.31, 3.85] | 0.00334 |
| Charlson Comorbidity Score on entry 5+ | 3.286 [1.89, 5.77] | 0.00003 |
| Mid | | |
| (Intercept) | 0.621 [0.11, 3.53] | 0.59359 |
| Age | 0.970 [0.95, 0.99] | 0.00204 |
| Community Controls | 1.220 [0.83, 1.80] | 0.31388 |
| PLWD LTC | 1.135 [0.76, 1.69] | 0.5338 |
| Rural | 0.911 [0.66, 1.26] | 0.57029 |
| Charlson Comorbidity Score on entry 1-2 | 1.481 [0.94, 2.41] | 0.10201 |
| Charlson Comorbidity Score on entry 3-4 | 2.232 [1.31, 3.85] | 0.00334 |
| Charlson Comorbidity Score on entry 5+ | 3.286 [1.89, 5.77] | 0.00003 |
| Late | | |
| (Intercept) | 0.621 [0.11, 3.53] | 0.59359 |
| Age | 0.970 [0.95, 0.99] | 0.00204 |
| Community Controls | 1.220 [0.83, 1.80] | 0.31388 |
| PLWD LTC | 1.135 [0.76, 1.69] | 0.5338 |
| Rural | 0.911 [0.66, 1.26] | 0.57029 |
| Charlson Comorbidity Score on entry 1-2 | 1.481 [0.94, 2.41] | 0.10201 |
| Charlson Comorbidity Score on entry 3-4 | 2.232 [1.31, 3.85] | 0.00334 |
| Charlson Comorbidity Score on entry 5+ | 3.286 [1.89, 5.77] | 0.00003 |

Polypharmacy analysis for males

| **Variable** | **Estimate** | **P value** |
| --- | --- | --- |
| All time periods | | |
| (Intercept) | 0.913 [0.26, 3.19] | 0.887 |
| Age | 0.998 [0.98, 1.01] | 0.781 |
| Community Controls | 0.796 [0.62, 1.03] | 0.080 |
| PLWD LTC | 1.075 [0.83, 1.39] | 0.576 |
| Rural | 1.135 [0.92, 1.40] | 0.232 |
| Charlson Comorbidity Score on entry 1-2 | 1.355 [1.01, 1.82] | 0.045 |
| Charlson Comorbidity Score on entry 3-4 | 1.756 [1.24, 2.48] | 0.001 |
| Charlson Comorbidity Score on entry 5+ | 1.205 [0.84, 1.74] | 0.316 |
| Early | | |
| (Intercept) | 0.371 [0.09, 1.44] | 0.153 |
| Age | 1.003 [0.99, 1.02] | 0.694 |
| Community Controls | 0.750 [0.57, 0.99] | 0.041 |
| PLWD LTC | 0.833 [0.64, 1.09] | 0.184 |
| Rural | 1.174 [0.94, 1.47] | 0.164 |
| Charlson Comorbidity Score on entry 1-2 | 1.167 [0.84, 1.62] | 0.354 |
| Charlson Comorbidity Score on entry 3-4 | 1.217 [0.84, 1.77] | 0.304 |
| Charlson Comorbidity Score on entry 5+ | 0.857 [0.57, 1.29] | 0.459 |
| Mid | | |
| (Intercept) | 0.509 [0.14, 1.84] | 0.304 |
| Age | 0.999 [0.98, 1.01] | 0.890 |
| Community Controls | 0.902 [0.69, 1.17] | 0.441 |
| PLWD LTC | 0.932 [0.72, 1.21] | 0.596 |
| Rural | 1.049 [0.85, 1.30] | 0.663 |
| Charlson Comorbidity Score on entry 1-2 | 1.660 [1.21, 2.29] | 0.002 |
| Charlson Comorbidity Score on entry 3-4 | 1.971 [1.38, 2.84] | 0.000 |
| Charlson Comorbidity Score on entry 5+ | 1.347 [0.92, 1.99] | 0.131 |
| Late | | |
| (Intercept) | 0.798 [0.22, 2.91] | 0.733 |
| Age | 0.996 [0.98, 1.01] | 0.570 |
| Community Controls | 0.817 [0.63, 1.06] | 0.135 |
| PLWD LTC | 0.969 [0.75, 1.26] | 0.811 |
| Rural | 1.073 [0.87, 1.33] | 0.522 |
| Charlson Comorbidity Score on entry 1-2 | 1.283 [0.94, 1.76] | 0.117 |
| Charlson Comorbidity Score on entry 3-4 | 1.379 [0.97, 1.97] | 0.078 |
| Charlson Comorbidity Score on entry 5+ | 0.894 [0.61, 1.32] | 0.573 |

Hyperpolypharmacy analysis for males

| **Variable** | **Estimate** | **P value** |
| --- | --- | --- |
| All time periods | | |
| (Intercept) | 0.454 [0.05, 3.77] | 0.46708 |
| Age | 0.971 [0.95, 0.99] | 0.01639 |
| Community Controls | 0.875 [0.57, 1.32] | 0.53012 |
| PLWD LTC | 0.957 [0.63, 1.45] | 0.83748 |
| Rural | 0.848 [0.60, 1.20] | 0.35017 |
| Charlson Comorbidity Score on entry 1-2 | 2.274 [1.12, 5.28] | 0.03566 |
| Charlson Comorbidity Score on entry 3-4 | 5.511 [2.67, 12.90] | 0.00002 |
| Charlson Comorbidity Score on entry 5+ | 6.632 [3.18, 15.65] | 0 |
| Early | | |
| (Intercept) | 0.454 [0.05, 3.77] | 0.46708 |
| Age | 0.971 [0.95, 0.99] | 0.01639 |
| Community Controls | 0.875 [0.57, 1.32] | 0.53012 |
| PLWD LTC | 0.957 [0.63, 1.45] | 0.83748 |
| Rural | 0.848 [0.60, 1.20] | 0.35017 |
| Charlson Comorbidity Score on entry 1-2 | 2.274 [1.12, 5.28] | 0.03566 |
| Charlson Comorbidity Score on entry 3-4 | 5.511 [2.67, 12.90] | 0.00002 |
| Charlson Comorbidity Score on entry 5+ | 6.632 [3.18, 15.65] | 0 |
| Mid | | |
| (Intercept) | 0.454 [0.05, 3.77] | 0.46708 |
| Age | 0.971 [0.95, 0.99] | 0.01639 |
| Community Controls | 0.875 [0.57, 1.32] | 0.53012 |
| PLWD LTC | 0.957 [0.63, 1.45] | 0.83748 |
| Rural | 0.848 [0.60, 1.20] | 0.35017 |
| Charlson Comorbidity Score on entry 1-2 | 2.274 [1.12, 5.28] | 0.03566 |
| Charlson Comorbidity Score on entry 3-4 | 5.511 [2.67, 12.90] | 0.00002 |
| Charlson Comorbidity Score on entry 5+ | 6.632 [3.18, 15.65] | 0 |
| Late | | |
| (Intercept) | 0.454 [0.05, 3.77] | 0.46708 |
| Age | 0.971 [0.95, 0.99] | 0.01639 |
| Community Controls | 0.875 [0.57, 1.32] | 0.53012 |
| PLWD LTC | 0.957 [0.63, 1.45] | 0.83748 |
| Rural | 0.848 [0.60, 1.20] | 0.35017 |
| Charlson Comorbidity Score on entry 1-2 | 2.274 [1.12, 5.28] | 0.03566 |
| Charlson Comorbidity Score on entry 3-4 | 5.511 [2.67, 12.90] | 0.00002 |
| Charlson Comorbidity Score on entry 5+ | 6.632 [3.18, 15.65] | 0 |
